# Supplementary figures and images for: Fundus autofluorescence features specific for EYS-associated retinitis pigmentosa
Source: PLoS One. 2025 Feb 19;20(2):e0318857. doi: 10.1371/journal.pone.0318857 (PMC11838866; doi:10.1371/journal.pone.0318857)

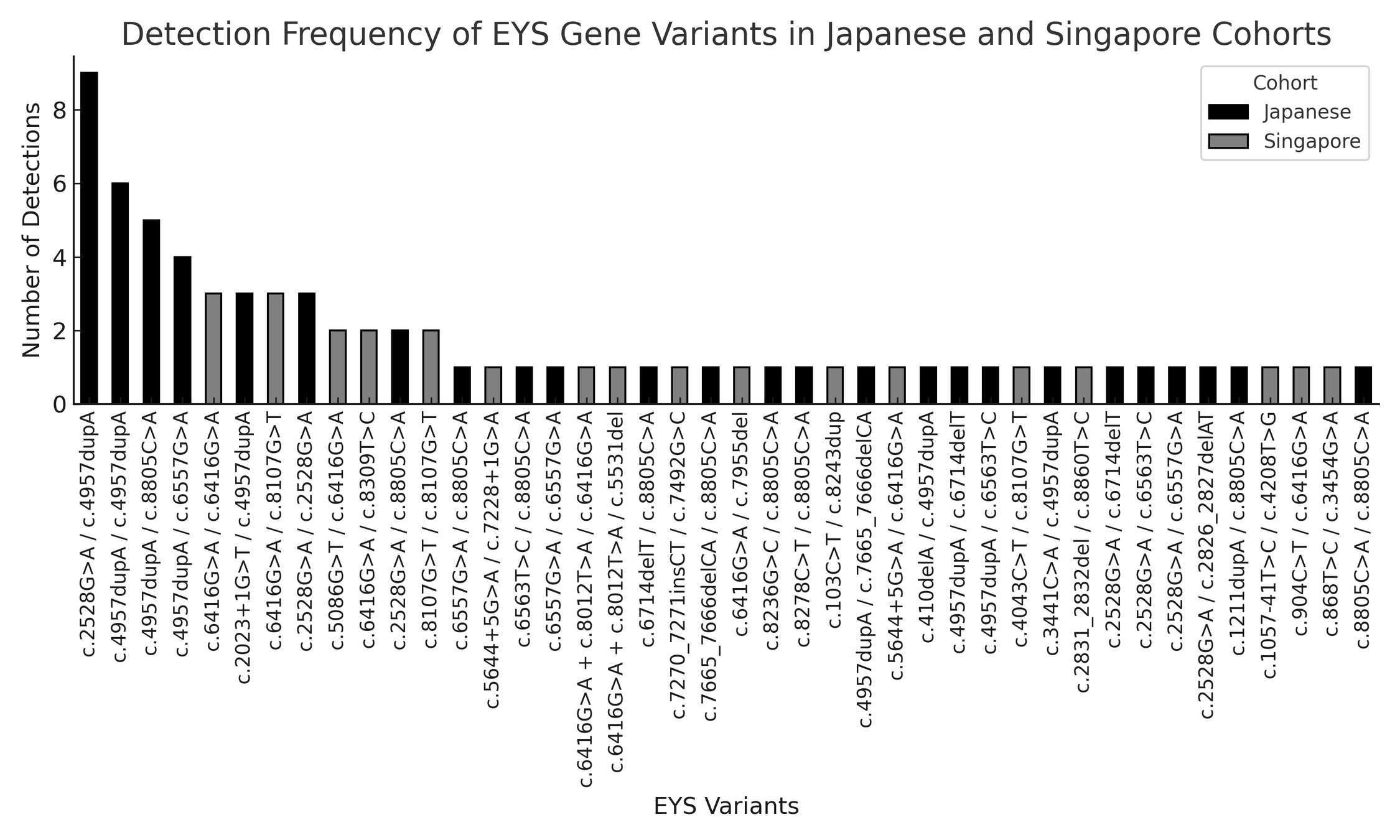

Supplement: S1 Fig — The bar chart shows the distribution of specific EYS gene variants identified in Japanese and Singaporean patient cohorts. The black bars represent the number of occurrences of each pathogenic variants in the Japanese cohort, while the gray bars represent the Singaporean cohort. Each bar corresponds to the number of times a specific variant or combination of variants was observed in the each population. (TIF) [file pone.0318857.s001.tif]

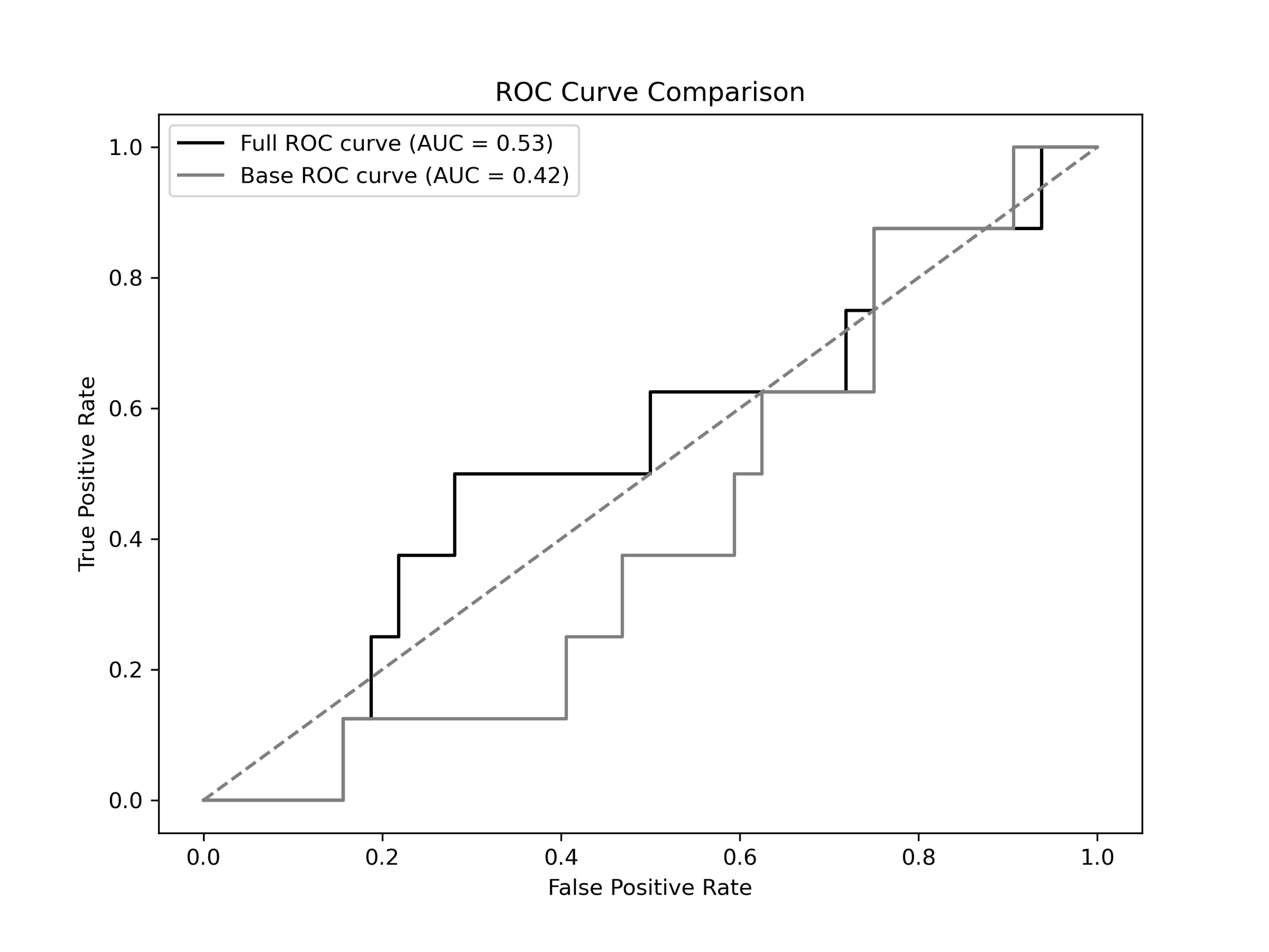

Supplement: S2 Fig — Non-EYS using Logistic Regression Models. S2 Fig shows the Receiver operating curve (ROC) curves comparing two logistic regression models for distinguishing between patients with EYS mutations and those without. The Full ROC curve (black line) represents the model using both the baseline parameters (age at imaging, age at onset, sex, Va) and additional fundus autofluorescence (FAF) parameters (crescent, foveal, infinity, broadband, robson, sectoral). The Base ROC curve (gray line) represents the model using only the baseline parameters. The area under the curve (AUC) for the Full ROC curve is 0.53, indicating a improved discriminative ability compared to the Base ROC curve, which has an AUC of 0.42. (TIF) [file pone.0318857.s002.tif]

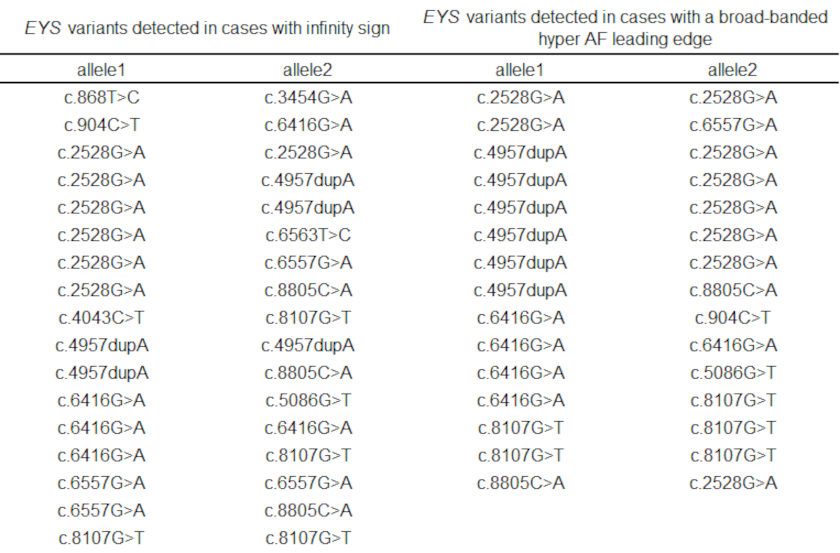

Supplement: S1 Table — (TIF) [file pone.0318857.s003.tif]
