# Supplementary material for: Fundus autofluorescence features specific for EYS-associated retinitis pigmentosa
Source: PLoS One. 2025 Feb 19;20(2):e0318857. doi: 10.1371/journal.pone.0318857 (PMC11838866; doi:10.1371/journal.pone.0318857)
Supplement: Attachment — Submitted filename: Response_to_reviewers.docx [file pone.0318857.s004.docx]

**January　17, 2025**
Tatsuya Inoue **Academic editor**
PLoS One

resubmission of Manuscript number PONE-D-24-48432

Dear Tatsuya Inoue,

Please find attached a revised version of our manuscript entitled “Novel Fundus Autofluorescence Features are Specific for *EYS*-Associated Retinitis Pigmentosa,” which we kindly request you to consider for publication as a *Research Article* in PLoS One.

We sincerely thank the reviewers for their thoughtful comments and their careful evaluation of our manuscript. We have made the suggested revisions to enhance the clarity and scientific validity of this study. Our detailed point-by-point responses are attached below. We have uploaded a marked-up copy of the manuscript that underlines the changes made to the original version. We believe that these revisions have further strengthened the manuscript and hope that it now meets the journal standards for publication.

All authors have approved the revised manuscript and agreed with its submission to PLoS One. The authors have declared that no competing interests exist.　All the funding or resources of support for this study were the Japan Society for the Promotion of Science (JSPS) KAKENHI, grant number 23K15929 to TK; the SingHealth Foundation grant number R1748/71/2020 to BJF; and the SingHealth - Duke-NUS Institute of Precision Medicine grant numbers 05/FY2020/EX/06-A41 and 05/FY2022/EX(SLP)/69-A131(b) to KJ, YMB, and WKL. Funding organizations had no role in the design or conduct of this study. There was no additional external funding received for this study.
Thank you for considering our manuscript for publication. We look forward to hearing from you.

Sincerely,

Beau James Fenner, MD, PhD
Department of Medical Retina, Singapore National Eye Centre, 11 Third Hospital Ave, 168751, Singapore
beaufenner@duke-nus.edu.sg
Tel: +65 9010 3000

Reviewer #1:
**Comment1**
The infinity sign and the broad banded hyperautofluorescent leading edge predicted EYS only in 17 of 74 eyes (23.0%) and in 15 of 74 eyes (20.3%), respectively. On the other hand, non-EYS RP eyes also showed these autofluorescence patterns in 6 of 126 eyes and 8 of 126 eyes, respectively. Although these percentages were low as compared with those for EYS-associated RP, non-EYE eyes are sum of multiple genes. What kind of genes caused the non-EYS RP eyes with positive findings of these autofluorescence patterns? If some specific genes were related to these autofluorescence, the information might be fruitful to understand the relationship of pathogenesis and autofluorescence patterns.
**Response1**
Thank you for your insightful comment. Upon further review of our data, we found that both the “infinity sign” and a broad-banded hyperautofluorescent (AF) leading edge also appeared at a relatively high frequency in *PRPH2*-associated RP compared with other non-*EYS* RP although it remains challenging to conclude whether these FAF features are truly characteristic of *PRPH2*-associated RP owing to its limited number. They share characteristic FAF findings possibly because both *EYS* and *PRPH2* are important for maintaining photoreceptor OS integrity. However, these two conditions have different inheritance patterns - *EYS* being Autosomal Recessive (AR) and *PRPH2* typically being Autosomal Dominant (AD). In the presence of an AD pedigree, the FAF findings might point towards *PRPH2*, while in sporadic or AR cases, they would point more towards *EYS*. Therefore, FAF as a biomarker would still be useful for *EYS*. We have added sentences reflecting these considerations to the Results (page 18 and 19, lines 200–204) and Discussion (pages 25 and 26, lines 301–313).

**Comment2**
(page 13, line 58) The abstract showed 100 patients with EYS and 100 without EYS, although the methods in the text stated 74 and 126, respectively.
**Response2**
Thank you for pointing this out to us. We have corrected the number of EYS and non-EYS (page5, lines 52 and 53).

**Comment3**
(page 13, line 60) The comma after ‘the infinity sign’ should be ‘and’.
**Response3**
Thank you for your comment. We have corrected this (page5, line 54).

**Comment4** (page 18, line 126) The word, ‘resulting’ should be ‘resulting in’.
**Response4**
Thank you for your comment. We have corrected this (page10, line 120).

**Comment5** (page 21, line 169 and Table 1) The ratio of Singaporeans and Japanese should be shown in Table 1.
**Response5**
Thank you for your comment. We have added the ratio of Singaporeans to Japanese to Table 1.

**Comment6** (page 21, line 173) The phrase in the parenthesis, ‘0.13 / 0.68…’ can be omitted because of statement in Table 1.
**Response6**
Thank you for your comment. We have omitted this (page14, line 178).

**Comment7** (Table 2) The total number is only 87. Were the pathogenic genes of the other 39 patients unknown? The authors should show all cases.
**Response7**
Thank you for pointing this out. We apologize for the confusion. If you simply add up the “number” column on the right side of the table, as you mentioned, it totals 87. However, for example, in the fourth row from the top, both RHO and RPGR are listed as seven, meaning that each gene accounts for seven cases, resulting in a total of 14. In other rows where multiple genes are listed, the number of cases for each gene is also reported separately. Consequently, if you consider the sum of each gene type on the left and the corresponding number on the right, the total number is 126. We have revised the term “number” in Table 2 to “Number of each gene” to make it clearer.

**Comment8** (Figure 2) P values for all groups should be shown in Figure 2.
**Response8**
Thank you for your comment. We have added P-values to Figure2.

**Comment9** (Table 4) What does ‘const’ mean?
**Response9**
Thank you for your comment. The “const” is a constant term obtained from the logistic regression results and does not hold any significance as an FAF parameter, so we have omitted it from Table 4.

**Comment10** (page 39) The reference, ‘Nakagawa S, …’ should be no. 19. Resultantly, the following no. 19-21 should be no. 20-22, respectively.
**Response10**
Thank you for pointing this out to us. We apologize for the errors in the reference numbers and list. We have corrected them accordingly(page27, lines 336 and 337; page34-35, lines 444-463).

Reviewer #2:
**Comment1**
Although there are various variants in EYS gene, it would be helpful to show how these mutations are specifically associated with the infinity sign.
**Response1**
Thank you for pointing this out to us. We summarized variants of EYS cases with “infinity sign” and a broad-banded hyperautofluorescent (AF) leading edge in the supplemental table. Among the EYS variants observed in cases presenting with an “infinity sign” or a broad-banded hyperAF leading edge, c.2528G>A and c.4957dupA appeared relatively frequently. However, these two variants are widely detected in general, and our current analysis did not detect a variant that was significantly correlated with FAF parameters. We have added sentences to the Results section(page18-19, lines 204-205) and Discussion section(page27, lines 329-333).

**Comment2**
Additionally, six cases of RP patients without EYS variants also exhibited the infinity sign. If possible, please identify the causative genes in these cases.
**Response2**
Thank you for your comments. Upon further review of our data, we found that the “infinity sign” also appeared at a relatively high frequency in *PRPH2*-associated RP compared with other non-*EYS* RP although it remains challenging to conclude whether “infinity sign” is truly characteristic of *PRPH2*-associated RP owing to its limited number. They share characteristic FAF findings possibly because both *EYS* and *PRPH2* are important for maintaining photoreceptor OS integrity. However, these two conditions have different inheritance patterns - *EYS* being Autosomal Recessive (AR) and *PRPH2* typically being Autosomal Dominant (AD). In the presence of an AD pedigree, “infinity sign” might point towards *PRPH2*, while in sporadic or AR cases, “infinity sign” would point more towards *EYS*. Therefore, FAF as a biomarker would still be useful for *EYS*. We have added sentences reflecting these considerations to the Results (page 18 and 19, lines 200–204) and Discussion (pages 25 and 26, lines 301–313).

**Comment3**
The autofluorescence patterns in RP would change during the disease progression. In this study, as noted in line 119, the cases include early-stage, late-stage, and cases with possible early cone dysfunction. However, the Discussion section does not address these changes. It is recommended to include a discussion of these changes.
**Response3**
Thank you for your comment. We have added the sentences to address this point in the Discussion section (page28, lines348-350).

**Comment4**
Line 250: Reference 19 is incorrect. Two references are included within Reference 18.Please verify whether the numbering of subsequent references is correct.
**Response4**
Thank you for pointing this out to us. We apologize for the errors in the reference numbers and list. We have corrected them accordingly(page27, lines 336 and 337; page34-35, lines 444-463).
